# Dietary Carotenoids and the Nervous System

**DOI:** 10.3390/foods4040698

**Published:** 2015-12-10

**Authors:** Billy R. Hammond

**Affiliations:** Department of Psychology, University of Georgia, Athens, GA 30602, USA; E-Mail: bhammond@uga.edu; Tel.: 706-542-4812

This issue of *Foods* is focused on the general topic of carotenoids within the nervous system. The focus is on the effects of the xanthophylls on the central nervous system (CNS), reflecting the majority of work in this area. This field of study is relatively new, despite knowing that carotenoids could be found, even within the brain itself, for nearly 40 years. The first report was in 1976 and described a patient taking high-dose beta-carotene as a treatment for erythropoietic protoporphyria. This patient had clearly detectable concentrations of beta-carotene within whole sections of the cerebrum [1]. The next notable contribution was by Cutler (1984) [2], who measured total carotenoids in the brain tissues of a variety of species (including humans), and argued that higher brain concentrations were linked to increased longevity when considered across species (a link between carotenoids and increased lifespan has also been argued within species ranging from drosophilia to human; [3,4]).

The first study that really quantified carotenoid within brain with any real specificity, however, was conducted by Craft *et al.* (2004) [5]. The Craft study analyzed carotenoid content in five elderly brains (dissecting various brain sections and distinguishing between gray and white matter) and found a seeming preference for xanthophylls in the human brain. This certainly made sense in that only two (of the 20 or so carotenoids circulating within blood) dietary carotenoids, lutein (L) and zeaxanthin (Z), are typically found in retina, and, there, they achieve millimolar concentrations (the highest accumulation of carotenoids in the body). The retina is central nervous system tissue and is widely regarded as “an approachable part of the brain” [6]. Not only are L and Z in ocular tissue at high amounts, their exclusive presence makes it clear that they are concentrated via some active mechanism. The brain, like the retina, appears to accumulate xanthophylls not simply through passive diffusion but actively. If this is so, then the implication is that these carotenoids have a role to play in the actual function of the brain itself.

As research has progressed, it has become clear that the carotenoids within the CNS do not play a singular role but appear to have a multitude of functions. Lutein, for instance, the dominant carotenoid in brain, has been described as a “work-horse molecule” [7]. The body often takes advantage of commonly available materials to serve a host of functions. For example, serotonin helps regulate not only gastrointestinal function and wound healing, but also dreaming, moods, and memory. Similarly, lutein appears to serve a surprising diversity of functions. One category, that the xanthophylls, in general, likely play within the CNS, appears to be simply prophylactic. The brain is largely fat and, of course, is very metabolically active and, hence, highly oxygenated. Carotenoids are known to inhibit lipid peroxidation. The brain is also clearly subject to inflammation, and carotenoids are known to be potent anti-inflammatory agents [8].

In addition to protection, however, the xanthophylls may serve other functions in brain tissue that range from epigenetic regulation to cellular communication [9,10]. On this front, the empirical work has outpaced basic research on mechanisms. We have some fairly convincing evidence that the xanthophylls influence the function of the central nervous system: Results ranging from temporal processing speed to cognition [11,12,13]. At present, however, we have very little understanding of how exactly the pigments achieve these changes.

It is perhaps surprising that it has taken us this long to study how *foods*, and the components of which they are composed, influence critically important tissues like the brain. Disciplines like psychology and nutrition have rarely interacted in the past. Although “we are what we eat” may be an oft-quoted axiom, it rarely translates into actual study. This appears to be changing. In 2005, Hammond and Wooten [14] found that macular xanthophylls were correlated with temporal processing speed, a measure known to be determined largely at the level of the visual cortex. As the authors noted, L and Z are “…providing some functional improvement unrelated to protection. L, for instance, in model systems…, has been shown to improve gap junction communication, which could improve cell to-cell communication within the nervous system. Thus, increasing L and Z intake could theoretically improve signaling efficiency throughout the visual system.”

In 2008, Johnson *et al.* [11] directly tested this idea and conducted a classic clinical trial (randomized, double-blind, placebo-controlled) on the efficacy of L, Z, and DHA on cognitive function of elderly women. That study found a statistically significant influence of these compounds on functions like verbal fluency and memory. The next pivotal studies in this area also emerged from the Jean Mayer Human Nutrition Research Center on Aging at Tufts University and were presented as conference abstracts in 2011 [15,16]This work confirmed Craft’s original finding of the predominance of xanthophylls in human brain and extended the work in a variety of ways: e.g., to infants and centenarians (the former helping to motivate the addition of xanthophylls to infant formula; the latter in collaboration with the Centenarian project at the University of Georgia).

Since 2011, the number of researchers studying the role of xanthophylls in brain function continues to increase (see, for example, [12,13]). As is often the case with highly applied science, the number of sponsors in this area has increased concomitantly (certainly suggesting more research to come). Thus far, research on how nutrition in general and xanthophylls specifically influence the central nervous system has been highly productive; so much so that it serves as an influential model for an emerging discipline which could be loosely described as nutritional neuroscience. This volume contains papers from many of the leaders in this inchoate area and covers topics ranging from eye to brain.
